# Minimally invasive surgery in emergency surgery: a WSES survey

**DOI:** 10.1186/s13017-022-00419-x

**Published:** 2022-03-18

**Authors:** Marco Ceresoli, Michele Pisano, Fikri Abu-Zidan, Niccolò Allievi, Kurinchi Gurusamy, Walt L. Biffl, Giovanni D. Tebala, Fausto Catena, Luca Ansaloni, Massimo Sartelli, Yoram Kluger, Gianluca Baiocchi, WSES MIS working group, Andreas Fette, Andreas Hecker, Andrey Litvin, Antonello Forgione, Ari Leppaniemi, Belinda De Simone, Boris Sakakushev, Casey R. Palmatier, Cino Bendinelli, Dimitris Damascos, Edoardo Picetti, Edward Tan, Elia Poiasina, Emmanouil Pikoulis, Enrico Cicuttin, Ernest E. Moore, George Velmahos, Gustavo Fraga, Harry Van Goor, Ian Civil, Imtiz Wani, Isidoro Di Carlo, Joseph Galante, Kjetil Søreide, Luca Degrate, Luigi Zorcolo, Marc De Moya, Marco Braga, Marco Cereda, Micheal Sugrue, Mircea Chirica, Nicola De Angelis, Philip F. Stahel, Rao Ivatury, Richard Ten Broek, Salomone Di Saverio, Solomon Gurmu Beka, Stefano Magnone, Yunfeng Cui, Zsolt J. Balogh, Micheal Dennis Kelly, Kenji Inaba, WSES MIS consortia, Federico Coccolini

**Affiliations:** 1 General and Emergency Surgery, School of Medicine and Surgery, Milano-Bicocca University, Via Pergolesi 33, 20900 Monza, Italy; 2 General and Emergency Surgery, ASST Papa Giovanni XXIII, Bergamo, Italy; 3 Department of Surgery, College of Medicine and Health Sciences, UAE University, Al-Ain, United Arab Emirates; 4 Division of Surgery and Interventional Science, University College London, London, UK; 5 Department of Therapy, I.M. Sechenov First Moscow State Medical University, Moscow, Russian Federation; 6 Scripps Medical Group, La Jolla, San Diego, CA USA; 7 Consultant Colorectal, Laparoscopic and Emergency Surgeon, Oxford University Hospitals NHSFT John Radcliffe Hospital, Headley Way, Headington, OX3 9DU Oxford UK; 8 General and Emergency Surgery Dept. Bufalini Hospital, Cesena, Italy; 9 General and Emergency Surgery, IRCCS San Matteo, University of Pavia, Pavia, Italy; 10 General and Emergency Surgery, Macerata Hospital, Macerata, Italy; 11 General Surgery, Rambam Medical Centre, Haifa, Israel; 12 General and Emergency Surgery, ASST Cremona; University of Brescia, Brescia, Italy; 13 Emergency Surgery, University of Pisa, Pisa, Italy; 14 Pediatric Surgery_Special Services (PS_SS) Weissach im Tal, Drosselstr 4, 71554 Weissach im Tal/FRG, Germany; 15 Universitäts klinikum Gießen und Marburg, Standort Gießen, Giessen, Germany; 16 Department of Surgical Disciplines, Immanuel Kant Baltic Federal University, Regional Clinical Hospital, Kaliningrad, Russia; 17 General Surgery, Niguarda Hospital, Milan, Italy; 18 Abdominal Center, Helsinki University Hospital and University of Helsinki, Helsinki, Finland; 19 Department of Emergency and Metabolic Minimally Invasive Surgery, Centre Hospitalier Intercommunal De Poissy/Saint Germain en Laye, Possy, France; 20 RIMU/Research Institute at Medical University of Plovdiv/UMHAT St George Plovdiv, Plovdiv, Bulgaria; 21 Henry Ford Professor and Edward J. Brickhouse Chairman| EVMS Surgery EVMS Medical Group, Norfolk, VA USA; 22 Endocrine and General Surgery, Conjoint Professor, University of Newcastle, Deputy Director of Trauma, John Hunter Hospital, Newcastle, Australia; 23 Consultant in General and Emergency Surgery,Royal Infirmary of Edinburgh, Honorary Clinical Senior Lecturer,Deanery of Clinical Sciences, College of Medicine and Veterinary Medicine, University of Edinburg, Edinburg, Scotland, UK; 24 Department of Anesthesia and Intensive Care, Parma University Hospital, Parma, Italy; 25 Chair Department of Emergency Medicine, Radboud UMC, Nijmegen, The Netherlands; 26 Professor of Surgery and Chairman of the 3rd Department of Surgery, Attikon General Hospital, National and Kapodistrian University of Athens (NKUA), Athen, Greece; 27 Ernest E Moore Shock Trauma Center, Denver Health, Dencer, CO USA; 28 John F. Burke Professor of Surgery, Harvard Medical School, Chief, Division of Trauma, Emergency Surgery, and Surgical Critical Care, Massachusetts General Hospital, Boston, MA USA; 29 Professor Associado do Departamento de Cirurgia, Coordenador da Disciplina de Cirurgia do Trauma, Coordenador do Escritório de Relações Internacionais Faculdade de Ciências Médicas (FCM) – Unicamp Campinas, São Paulo, SP Brasil; 30 Head of Surgical Research Laboratory, Chair Theme Reconstructive and Regenerative Medicine, Radbouc UMC, Nijmegen, The Netherlands; 31 Surgery, Auckland City Hospital, Aucland, New Zealand; 32 Government Gousia Hospital, Srinagar, Kashmir India; 33 Professor of Surgery, Department of Surgical Sciences and Advanced Technologies, University of Catania, General Surgery Cannizzaro Hospital, Catania, Italy; 34 Medical Director, Perioperative Services,Trauma Medical Director, Professor of Surgery, University of California, Davis, Sacramento, CA USA; 35 Department of Gastrointestinal Surgery | Stavanger University Hospital - Department of Clinical Medicine, University of Bergen, Bergen, Norway; 36 General Surgery, ASST Monza, Monza, Italy; 37 General Surgery, University of Cagliari, Cagliari, Italy; 38 Professor and Chief of Trauma/Acute Care Surgery, Lunda/Aprahamian Chair Medical College of Wisconsin/Froedtert Trauma Center, Milwaukee, WI USA; 39 General Surgery, Letterkenny Hospital, Donegal, Ireland; 40 General Surgery, Centre Hospitalier Universitaire de Grenoble, Grenoble, France; 41 Unit of Digestive and HPB Surgery, CARE Department Henri Mondor University Hospital (AP-HP), Faculty of Medicine, University of Paris Est, Créteil, France; 42 The Medical Center of Aurora and Spalding Rehabilitation Hospital, Aurora, CO USA; 43 Chief, Trauma, Critical Care, Emergency Surgery, VCU Health System, Richmond, VA USA; 44 Surgery, Radbouc UMC, Nijmegen, The Netherlands; 45 Director of General Surgery Department, ASUR Marche, AV5, Hospital of San Benedetto del Tronto (AP) - Sapienza University, Rome, Italy; 46 Consultant Emergency Surgery, Professional Specialist at Ethiopian Air Force Hospital, President of Ethiopian Emergency Surgery Association, Bishoftu, Oromia Ethiopia; 47 Department of Surgery, Tianjin Nankai Hospital, Nankai Clinical School of Medicine, Tianjin Medical University, Tianjin, China; 48 Department of Traumatology, John Hunter Hospital and University of Newcastle, Newcastle, NSW Australia; 49 MedAlliance, Albury, NSW Australia; 50 Professor and Vice Chair of Surgery, LAC+USC Medical Center, University of Southern California, Los Angeles, CA USA

**Keywords:** Minimally invasive surgery, Emergency surgery, Laparoscopy, Survey

## Abstract

**Background:**

The diffusion of minimally invasive surgery in emergency surgery still represents a developing challenge. Evidence about the use of minimally invasive surgery shows its feasibility and safety; however, the diffusion of these techniques is still poor. The aims of the present survey were to explore the diffusion and variations in the use of minimally invasive surgery among surgeons in the emergency setting.

**Methods:**

This is a web-based survey administered to all the WSES members investigating the diffusion of minimally invasive surgery in emergency. The survey investigated personal characteristics of participants, hospital characteristics, personal confidence in the use of minimally invasive surgery in emergency, limitations in the use of it and limitations to prosecute minimally invasive surgery in emergency surgery. Characteristics related to the use of minimally invasive surgery were studied with a multivariate ordinal regression.

**Results:**

The survey collected a total of 415 answers; 42.2% of participants declared a working experience > 15 years and 69.4% of responders worked in tertiary level center or academic hospital. In primary emergencies, only 28,7% of participants declared the use of laparoscopy in more than 50% of times. Personal confidence with minimally invasive techniques was the highest for appendectomy and cholecystectomy. At multivariate ordinal regression, a longer professional experience, the use of laparoscopy in major elective surgery and bariatric surgery expertise were related to a higher use of laparoscopy in emergency surgery.

**Conclusions:**

The survey shows that minimally invasive techniques in emergency surgery are still underutilized. Greater focus should be placed on the development of dedicated training in laparoscopy among emergency surgeons.

**Supplementary Information:**

The online version contains supplementary material available at 10.1186/s13017-022-00419-x.

## Introduction

Laparoscopy still represents the cornerstone of minimally invasive surgery (MIS); after 80 years of surgery performed by laparotomy, on September 12, 1980, the first laparoscopic appendectomy was performed, followed by a rapid expansion of this technique. The first laparoscopic cholecystectomy was performed between 1985 and 1987 in different parts of the world. From this date, all the abdominal quadrants and organs have been the targets for laparoscopic procedures, mostly in elective cases [1–4]. The pathway for the acceptance of elective laparoscopy has not been straightforward as reported for cholecystectomy [5]. However, technical limitations became the trigger for pioneers and industries to allow safer and simpler surgical maneuvers by improvement in visualization, dissectors and sealing devices [6]. Finally, also oncological issues have been investigated, in the last two decades, with good quality studies [7, 8].

Reasons for MIS development are heterogeneous and, in some way, interconnected: reduction of patient’s surgical stress, reduced postoperative pain, early return to regular activities, cosmetic advantages, the human innate propensity to innovation among surgeons, industries and economic factors. All these issues can be smoothly managed in elective cases and counterbalanced with patient safety, oncological issues, operating room occupancy, etc.

When we move to MIS in Emergency Surgery, the amount of available literature decreases in number and above all in quality, resulting in lot of uncertainty. According to Surgical Societies guidelines and large retrospective studies with literature reviews, laparoscopic appendectomy, cholecystectomy and gastric and duodenal ulcer repair are well accepted emergency surgical procedures. However, their diffusion, even in the same hospital, can be influenced by insufficient expertise that may correlate with hospital organizational model. Other surgical procedures such as laparoscopic treatment of small bowel occlusion, bowel resection for acute diverticulitis, are becoming more frequent but they are still not routinely suggested [9–14].

In a recent report of a large observational study from UK, laparoscopy is adopted in less than 20% of major surgeries in emergency [15].

These difficulties of diffusion of minimally invasive surgery in emergency setting could be attributed to several reasons, i.e., more complexity when compared to elective surgery, sicker patients, higher level of diagnostic uncertainty, no regular day and week working hours, organizational issue, the lack of a dedicated surgical training and not homogeneous surgical and team skills.

The aims of this survey were to explore whether there are variations in the use of minimally invasive surgery among surgeons in the emergency setting and if there were variations, the potential determinants of these variations.

## Methods

### Study design

This is cross-sectional study, which was performed during the period of March 21st 2021 to August 14th 2021 among the members of the World Society of Emergency Surgery.

### Sample size

An invitation to participate to the survey was sent for all the members of the World Society of Emergency Surgery (WSES) through their email with the invite to extend the survey to all their colleagues. Sample size calculation is not required in this situation because all subjects were approached.

### Questionnaire design

The on-line questionnaire is shown in Additional file 1. The design of the questionnaire was developed according to the published recommendations for the development and implementation of web-based surveys (CHERRIES) [16, 17] adopting the Google form tool (Alphabet Inc., Mountain View, CA, USA). It was written in English by a steering committee nominated by the WSES board. The final questionnaire was endorsed by the WSES board.

The self-administered questionnaire was developed in 5 sections:

1. Personal characteristics,
2. Hospital characteristics,
3. Personal confidence in the use of minimally invasive surgery in emergency surgery,
4. Limitations of the use of minimally invasive surgery in emergency surgery,
5. Limitations to prosecute minimally invasive surgery in emergency surgery.

The countries of provenience were grouped into the six WHO regions (African region, American region, East Mediterranean Region, European Region, Southeast Asian region and West Pacific Region). Surgeries were divided into three categories: major elective abdominal surgery, primary emergency and secondary emergency (re-intervention after elective surgery). Minimally invasive emergency surgery interventions were further classified into four categories based on increasing difficulty (grade 1: appendectomy and cholecystectomy; grade 2: peptic ulcer perforation repair and adhesiolysis; grade 3: colonic resection for acute diverticulitis; grade 4: secondary emergencies). Questions about participants’ perceptions were ranked with response options from 0 to 5.

### Validity and piloting

The study has mainly depended on surface validity. Content validity depended on the knowledge and experience of experts. The questionnaire was not piloted. Linguistic clarity was reviewed by 3 international experts from 2 different countries. The experts have different mother tongue languages including English and Italian which assured us that the language used in the questionnaire was clear and not ambiguous for the international participants.

### Distribution of the survey and data collection

The invitation to the survey was distributed through WSES web during the period of March 21st 2021 to August 14th 2021. Four reminders were sent to the WSES members email list. Data were collected directly and stored through the website into an on-line database which was protected by a secure password. No incentives for participation were given.

### Ethical considerations

The participation to the survey was voluntary and anonymous; an email address of each participant was used for invitation but no personal identifiers were collected. Confidentiality of respondents and their choices were secured. An ethical approval was not needed.

### Statistical analysis

The results of the survey were shown as median along with Interquartile range for the continuous variable and percentages for categorical variables. A ordinal logistic regression model was calculated to investigate the role of respondent and hospital characteristics in the self-reported rate of use of laparoscopy in primary emergencies. Statistics were calculated with SPSS (IBM Corp. Released 2020. IBM SPSS Statistics for Windows, Version 27.0. Armonk, NY: IBM Corp).

## Results

The survey collected a total of 415 answers from 67 countries: the majority come from the European (66.5%) and American regions (17.8%). Median age of participants was 43 (37–52) and 85.8% were men; 35.4% of participants declared a working experience > 6 years and 42.2% > 15 years. Most responders (69.4%) worked in tertiary level center or academic hospital and 29.4% were dedicated to emergency surgery for more than 50% of the time. Table 1 shows the complete details of participants.

**Table 1:** Characteristics of survey respondents

	Median (IQR)	*N*	%
Age	43 (37–52)		
Sex			
Male		356	85.8
Female		59	14.2
WHO Area			
African region		11	2.7
American Region		74	17.8
Southeast Asian Region		18	4.3
European Region		276	66.5
East Mediterranean Region		13	3.1
West Pacific Region		23	5.5
Professional experience			
Resident		12	2.9
0–5 years		81	19.5
6–15 years		147	35.4
> 15 years		175	42.2
Self-declared Expertise			
General Surgery	4 (3–5)		
Emergency and Trauma Surgery	4 (3–5)		
Colorectal Surgery	3 (2–4)		
Upper GI	3 (2–4)		
HPB	2 (1–4)		
Endocrine Surgery	2 (1–3)		
Bariatric Surgery	1 (0–2)		
Kind of Hospital			
Private Hospital		104	25.1
Public Hospital		311	74.9
Hospital Level			
First level – rural		21	5.1
Second level		106	25.5
Academic—referral hospital		288	69.4
presence of Emergency department		392	94.5
dedicated Emergency surgery unit		175	42.2
Time dedicated to emergency surgery			
0%		4	1.0
1–25%		142	34.2
26–50%		147	35.4
> 50%		122	29.4

**Fig. 1:**
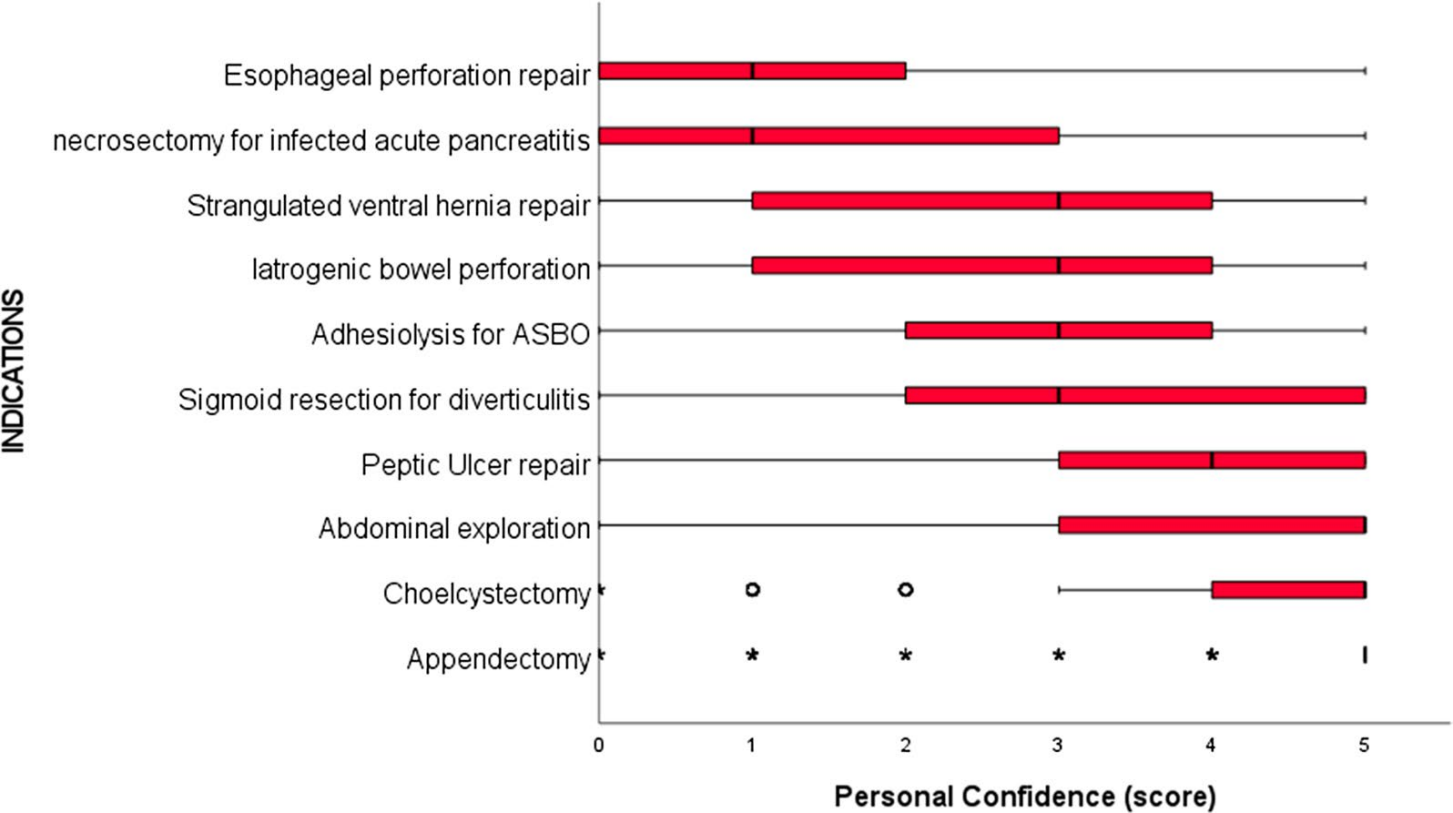
Box-plot of self-declared personal confidence in MIS for primary emergencies; personal confidence is graded from 0 (no confidence) to 5 (maximum confidence)

**Fig. 2:**
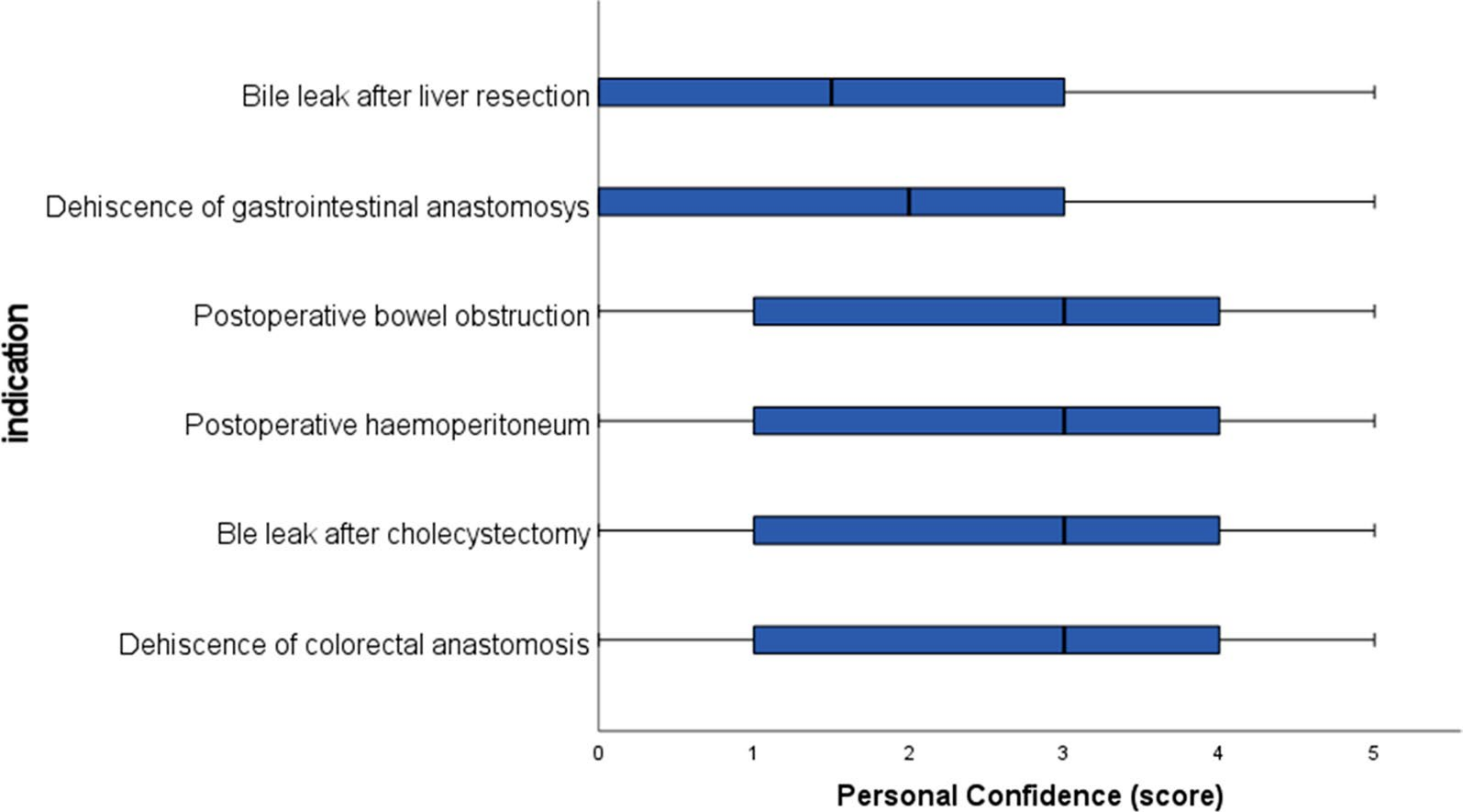
Box-plot of self-declared personal confidence in MIS for secondary emergencies; personal confidence is graded from 0 (no confidence) to 5 (maximum confidence)

Table 2 shows the self-declared rate of use of laparoscopy and robotic in elective and emergency surgery: laparoscopy was used in major elective abdominal surgery in more than 50% of patients in half of participants (52%); in primary emergencies, only a quarter of participants (28,7%) declared the use of laparoscopy in more than 50% of patients. The personal confidence with minimally invasive techniques was the highest for appendectomy, cholecystectomy and abdominal exploration (median score 5) while it was lowest for necrosectomy in infected acute pancreatitis and esophageal perforations (median score 1). Complete results are shown in Figs. 1 and 2.

**Table 2:** Self-declared rate of use of laparoscopy and robotic in elective and emergency surgery

	N	%
*Self-declared use of minimally invasive surgery—laparoscopy*		
Laparoscopy in major elective surgery		
Never	18	4.3
0–25%	80	19.3
26–50%	101	24.3
More than 50%	216	52.0
Laparoscopy in primary surgical emergencies		
Never	18	4.3
0–25%	102	24.6
26–50%	176	42.4
More than 50%	119	28.7
Laparoscopy in secondary surgical emergencies		
Never	47	11.3
0–25%	181	43.6
26–50%	107	25.8
More than 50%	80	19.3
*Self-declared use of minimally invasive surgery—robotic*		
Robotic in major elective surgery		
Never	346	83.4
0–25%	46	11.1
26–50%	14	3.4
More than 50%	9	2.2
Robotic in primary surgical emergencies		
Never	400	96.4
0–25%	11	2.7
26–50%	2	0.5
More than 50%	2	0.5
Robotic in secondary surgical emergencies		
Never	398	95.9
0–25%	14	3.4
26–50%	1	0.2
More than 50%	2	0.5

Table 3 shows the results about the limiting factors in performing minimally invasive emergency surgery: technical skills, technical availability, nursing skills, night-time operation and estimated prolonged duration of surgery were not perceived as great limitations for grades 1 to 3 of surgical difficulty. Among patient-related limiting factors, the condition of shock (hemodynamic instability) was a limiting factor across all grades (median scores of 3 for grade 1 surgical difficulty and 4 for the remaining grades). Among the intraoperative limiting factors, the deterioration of clinical conditions during surgery and unclear visualization of the anatomy was the main reasons why minimally invasive surgery was abandoned (median scores of4 across all grades of severity). Detailed results are shown in Table 3.

**Table 3:** Limiting factors in performing minimally invasive emergency surgery

	Appendicitis, Cholecystitis (Grade 1)	Perforation of Gastric and Duodenal Ulcer, Bowel Obstruction due to peritoneal adhesions (Grade 2)	Colon resection for Hinchey 3 and 4 Acute Diverticulitis (Grade 3)	Dehiscence of intestinal, colorectal, gastrointestinal anastomosis; bile leak after cholecystectomy, bile leak after liver resection, postoperative hemoperitoneum, postoperative intestinal obstruction (Grade 4)
	Median (IQR)	Median (IQR)	Median (IQR)	Median (IQR)
Limiting factors				
Your own surgical skills	1 (0–4)	2 (0–3)	2 (1–4)	2 (1–4)
Nursing skills	1 (0–3)	1 (0–3)	1 (0–3)	1 (0–3)
Night-time operation	1 (0–3)	2 (0–3)	2 (1–4)	2 (1–4)
Technology availability	2 (0–4)	2 (0–4)	2 (0–4)	2 (0–4)
Estimated prolonged surgical duration	1 (0–3)	2 (1–3)	2 (1–4)	3 (1–4)
Patient-related limiting factors				
Shock condition	3 (1–5)	4 (2–5)	4 (3–5)	4 (3–5)
Age	1 (0–3)	1 (0–3)	2 (0–3)	2 (0–3)
ASA score	2 (1–3)	2 (1–4)	3 (1–4)	3 (1–4)
APACHE score	2 (1–3)	2 (1–4)	3 (1–4)	3 (1–4)
P-POSSUM	2 (1–3)	2 (1–3)	2 (1–4)	3 (1–4)
ACSNSQUIP Surgical Risk	2 (1–3)	2 (1–3)	2 (1–4)	3 (1–4)
Previous abdominal surgery	2 (1–4)	3 (1–4)	3 (2–4)	3 (2–4)
Intraoperative limiting factors				
Duration of the surgical procedure	2 (1–3)	2 (1–4)	3 (1–4)	3 (1–4)
Bleeding	3 (2–4)	3 (1–4)	3 (2–4)	3 (1–4)
Unclear/suboptimal visualization of anatomical structures	4 (2–5)	4 (2–5)	4 (2–5)	4 (3–5)
Bowel perforation	3 (1–4)	3 (1–4)	3 (2–4)	3 (2–4)
Intraoperative clinical deterioration	4 (2–5)	4 (2–5)	4 (3–5)	4 (3–5)

The multivariate ordinal logistic regression identified factors independently correlated with the use of laparoscopy in primary emergencies. A longer professional experience (OR 1.54, 95% CI 1.07–2.21 per additional year of surgical experience), the use of laparoscopy in major elective surgery (OR 4.13, 95% CI 3.11–5.47) and bariatric surgery expertise (OR 1.37, 95%CI 1.17–1.60) were significantly related to a higher use of laparoscopy in emergency surgery. Surgeons subspecializing in colorectal surgery (OR 0.77, 95%CI 0.62–0.95) and endocrine surgery (OR 0.75, 95%CI 0.63–0.90) used less laparoscopy in emergency procedures, while those subspecializing in bariatric surgery (OR 1.37, 95%CI 1.17–1.60) used more laparoscopy in emergency procedures.

## Discussion

The results of the present survey show that the diffusion of minimally invasive techniques in emergency surgery is still quite limited. The confidence of surgeon in minimally invasive techniques is higher for simple surgical interventions as appendectomy, cholecystectomy and abdominal exploration but decreases progressively with the increasing difficulty of surgery. The characteristics related to a higher use of laparoscopy in primary emergencies are longer personal surgical experience, extensive use of laparoscopy in major elective abdominal surgery, and bariatric surgical expertise.

According to the literature, laparoscopy is used in less than 20% of major emergency operations: the results of a recent research study from the National Emergency Laparotomy Audit (NELA) of England and Wales described that only 14.6% of cases were approached by laparoscopy with a conversion rate of 46.4% [15]. A research study from the USA reported an higher proportion of minimally invasive surgery in emergency general surgery (69.4%), but the majority of interventions were appendectomy and cholecystectomy: the proportion of major abdominal surgery in emergency performed with minimally invasive techniques was less than 20% [18]. Regarding major colorectal emergency surgery, several reports describe feasibility and safety; moreover, the promotion of the use of MIS is proved by lot of didactic articles [19–21]; however, in a large report, the proportion of patients treated with MIS was only 5.66% [19]. Data available in literature and the results of the present survey highlight an important need to improve the safe and effective use of minimally invasive techniques in emergency surgery.

Among the characteristics of surgeons who answered to the survey the main factors related to a higher and more significant diffusion of laparoscopy in emergency surgery were the longer personal experience and the use of laparoscopy in elective surgery: these data highlight the important role of personal skills in increasing the use of minimally invasive techniques. Similarly, expertise in bariatric surgery and prevalent use of laparoscopy in major abdominal surgery were directly related to the use of laparoscopy in emergency surgery. Literature data and the results of our survey suggest that there is plenty of room for improvement in the safe and effective use and the diffusion of minimally invasive techniques also in emergency surgery. Dedicated training in emergency laparoscopic surgery and initiatives of continuing professional development may be beneficial in order to be able to offer the advantages of mini-invasive approaches to a larger number of patients also in emergency.

Moreover, our data offer the opportunity to reflect on which is the best organizational model for emergency surgery.

A surgeon with more developed skills in elective surgery and more experienced in elective laparoscopic surgery is more prone to use laparoscopic surgery also in primary emergencies. On the contrary, emergency and Trauma surgery usually requires dedicated teams with specific skills [22, 23] that may not include minimally invasive techniques.

Only 29.4% of surgeons who answered the survey declared to be dedicated to emergency surgery for more than 50% of their time. However, a longer time dedicated to emergency surgery was not significantly related to a lower use of laparoscopy in primary emergency at the multivariate analysis, showing a very complex interaction with several other characteristics as personal experience and personal expertise. Almost exclusive emergency surgery practice is not associated with lack of confidence with MIS, but extensive elective laparoscopic experience is for sure a positive factor.

Despite these considerations, data about the limiting factors to the use of minimally invasive techniques in emergency surgery show that a surgeons' perception of their surgical skills was not considered to be a limiting factor (Table 3). Similarly, the night-time, the nursing skills and the technology availability do not seem to be major limiting factors. This may reflect the intrinsic ability of surgeons to adapt to the circumstances and their strong commitment to improvement in any environmental situation. Among patients’ conditions, the only factors that seem to be limiting factors in the use of minimally invasive surgery in emergency surgery is the shock condition, while age and high predicted morbidity and mortality according to the most common clinical scores as ASA, P-POSSUM and APACHE II are perceived as important limiting factors for difficult surgeries (median score 3).

The results of the present survey should be interpreted with caution at the light of some considerations. First of all, the relatively small number of respondents represents a highly selected population of surgeons; in fact, most respondents works in academic and tertiary hospitals mostly from the European and the American WHO regions. Moreover, the majority of respondents are surgeons with a particular interest in emergency and trauma surgeon with an active participation or an affiliation to a scientific society. It should be also noticed that the survey was focused mostly on abdominal surgery and no considerations can be drawn about other surgical specialities (Table 4).

**Table 4:** Multivariate ordinal regression on use of MIS in primary emergencies

Variable	OR	95% confidence interval		*p* value
		Lower	Upper	
Sex				
Male	1			
Female	0.715	0.400	1.277	0.257
Age (+ 1)	0.982	0.952	1.012	0.238
WHO Region				
West Pacific Region	1.789	0.413	7.738	0.437
East Mediterranean Region	1.299	0.249	6.775	0.756
Europe	3.078	0.875	10.826	0.080
Southeast Asian Region	0.906	0.181	4.522	0.904
American Region	3.409	0.921	12.622	0.066
African Region	1			
Kind of Hospital				
Public hospital	1			
Private Hospital	1.193	0.740	1.924	0.470
Hospital Level				
First level hospital	0.524	0.208	1.320	0.171
Second level hospital	0.826	0.319	2.138	0.693
Academic Hospital	1			
Presence of Emergency department	1.401	0.601	3.263	0.435
Presence of Acute care service /unit	0.795	0.508	1.245	0.316
**Years of professional experience (+ 1)**	**1.544**	**1.074**	**2.219**	**0.019**
Personal expertise				
General surgery	1.193	0.878	1.621	0.258
Emergency and trauma surgery	1.130	0.885	1.442	0.327
**Colorectal expertise**	**0.770**	**0.621**	**0.955**	**0.017**
Upper GI	1.060	0.850	1.323	0.604
HPB	0.997	0.833	1.194	0.978
**Endocrine surgery**	**0.756**	**0.636**	**0.900**	**0.002**
**Bariatric surgery**	**1.370**	**1.171**	**1.603**	**< 0.001**
**Use of laparoscopy in elective surgery**	**4.130**	**3.116**	**5.474**	**< 0.001**
Time dedicated to emergency surgery	1.256	0.951	1.658	0.108

Significant variables are marked in bold

For these reasons—this selection bias and the relatively small number of participants from developing countries and smaller hospitals—the present survey may not be an accurate description of the real-world uptake of minimally invasive surgery techniques in emergency surgery. The WHO region and technology availability were not significantly related to an increased or decreased use of laparoscopy in emergency surgery; however, the confidence intervals were very wide indicating the uncertainty about this issue. This suggests the need for further research in order to describe the role of availability of technology in smaller hospitals.

## Conclusions

In conclusion, the present survey shows that minimally invasive techniques in emergency surgery are still underutilized by a large proportion of surgeons. Among the factors related to a larger adoption of minimally invasive techniques, the most important are the adoption of laparoscopy in elective surgery and increased surgical experience. In order to improve the uptake of minimally invasive technique in the emergency setting, greater focus should be placed on the development of dedicated training in laparoscopy.

## Supplementary Information

**Additional file 1**: Survey's questionnaire.

**Additional file 2**: Members of the WSES MIS Consortia.

## Data Availability

Data are available on request to the corresponding author.

## Abbreviations

MIS: Minimally Invasive Surgery
WSES: World Society of Emergency Surgery
WHO: World Health Organization
ASA: American Association of Anesthesiology
