# Correction: Minimally invasive surgery in emergency surgery: a WSES survey

**DOI:** 10.1186/s13017-022-00451-x

**Published:** 2022-08-29

**Authors:** Marco Ceresoli, Michele Pisano, Fikri Abu-Zidan, Niccolò Allievi, Kurinchi Gurusamy, Walt L. Biffl, Giovanni D. Tebala, Fausto Catena, Luca Ansaloni, Massimo Sartelli, Yoram Kluger, Gianluca Baiocchi, WSES MIS working group, Andreas Fette, Andreas Hecker, Andrey Litvin, Antonello Forgione, Ari Leppaniemi, Belinda De Simone, Boris Sakakushev, Casey R. Palmatier, Cino Bendinelli, Dimitris Damaskos, Edoardo Picetti, Edward Tan, Elia Poiasina, Emmanouil Pikoulis, Enrico Cicuttin, Ernest E. Moore, George Velmahos, Gustavo Fraga, Harry Van Goor, Ian Civil, Imtiz Wani, Isidoro Di Carlo, Joseph Galante, Kjetil Søreide, Luca Degrate, Luigi Zorcolo, Marc De Moya, Marco Braga, Marco Cereda, Micheal Sugrue, Mircea Chirica, Nicola De Angelis, Philip F. Stahel, Rao Ivatury, Richard Ten Broek, Salomone Di Saverio, Solomon Gurmu Beka, Stefano Magnone, Yunfeng Cui, Zsolt J. Balogh, Micheal Dennis Kelly, Kenji Inaba, WSES MIS consortia, Federico Coccolini

**Affiliations:** 1 General and Emergency Surgery, School of Medicine and Surgery, Milano-Bicocca University, Via Pergolesi 33, 20900 Monza, Italy; 2 General and Emergency Surgery, ASST Papa Giovanni XXIII, Bergamo, Italy; 3 Department of Surgery, College of Medicine and Health Sciences, UAE University, Al-Ain, United Arab Emirates; 4 Division of Surgery and Interventional Science, University College London, London, UK; 5 Department of Therapy, I.M. Sechenov First Moscow State Medical University, Moscow, Russian Federation; 6 Scripps Medical Group, La Jolla, San Diego, CA USA; 7 Consultant Colorectal, Laparoscopic and Emergency Surgeon, Oxford University Hospitals NHSFT John Radcliffe Hospital, Headley Way, Headington, OX3 9DU Oxford UK; 8 General and Emergency Surgery Dept. Bufalini Hospital, Cesena, Italy; 9 General and Emergency Surgery, IRCCS San Matteo, University of Pavia, Pavia, Italy; 10 General and Emergency Surgery, Macerata Hospital, Macerata, Italy; 11 General Surgery, Rambam Medical Centre, Haifa, Israel; 12 General and Emergency Surgery, ASST Cremona; University of Brescia, Brescia, Italy; 13 Emergency Surgery, University of Pisa, Pisa, Italy; 14 Pediatric Surgery_Special Services (PS_SS) Weissach im Tal, Drosselstr 4, 71554 Weissach im Tal/FRG, Germany; 15 Universitäts klinikum Gießen und Marburg, Standort Gießen, Giessen, Germany; 16 Department of Surgical Disciplines, Immanuel Kant Baltic Federal University, Regional Clinical Hospital, Kaliningrad, Russia; 17 General Surgery, Niguarda Hospital, Milan, Italy; 18 Abdominal Center, Helsinki University Hospital and University of Helsinki, Helsinki, Finland; 19 Department of Emergency and Metabolic Minimally Invasive Surgery, Centre Hospitalier Intercommunal De Poissy/Saint Germain en Laye, Possy, France; 20 RIMU/Research Institute at Medical University of Plovdiv/UMHAT St George Plovdiv, Plovdiv, Bulgaria; 21 Henry Ford Professor and Edward J. Brickhouse Chairman| EVMS Surgery EVMS Medical Group, Norfolk, VA USA; 22 Endocrine and General Surgery, Conjoint Professor, University of Newcastle, Deputy Director of Trauma, John Hunter Hospital, Newcastle, Australia; 23 Consultant in General and Emergency Surgery, Royal Infirmary of Edinburgh, Honorary Clinical Senior Lecturer, Deanery of Clinical Sciences, College of Medicine and Veterinary Medicine, University of Edinburg, Edinburg, Scotland, UK; 24 Department of Anesthesia and Intensive Care, Parma University Hospital, Parma, Italy; 25 Chair Department of Emergency Medicine, Radboud UMC, Nijmegen, The Netherlands; 26 Professor of Surgery and Chairman of the 3rd Department of Surgery, Attikon General Hospital, National and Kapodistrian University of Athens (NKUA), Athen, Greece; 27 Ernest E Moore Shock Trauma Center, Denver Health, Dencer, CO USA; 28 John F. Burke Professor of Surgery, Harvard Medical School, Chief, Division of Trauma, Emergency Surgery, and Surgical Critical Care, Massachusetts General Hospital, Boston, MA USA; 29 Professor Associado do Departamento de Cirurgia, Coordenador da Disciplina de Cirurgia do Trauma, Coordenador do Escritório de Relações Internacionais Faculdade de Ciências Médicas (FCM) – Unicamp Campinas, São Paulo, SP Brasil; 30 Head of Surgical Research Laboratory, Chair Theme Reconstructive and Regenerative Medicine, Radbouc UMC, Nijmegen, The Netherlands; 31 Surgery, Auckland City Hospital, Aucland, New Zealand; 32 Government Gousia Hospital, Srinagar, Kashmir India; 33 Professor of Surgery, Department of Surgical Sciences and Advanced Technologies, University of Catania, General Surgery Cannizzaro Hospital, Catania, Italy; 34 Medical Director, Perioperative Services, Trauma Medical Director, Professor of Surgery, University of California, Davis, Sacramento, CA USA; 35 Department of Gastrointestinal Surgery | Stavanger University Hospital - Department of Clinical Medicine, University of Bergen, Bergen, Norway; 36 General Surgery, ASST Monza, Monza, Italy; 37 General Surgery, University of Cagliari, Cagliari, Italy; 38 Professor and Chief of Trauma/Acute Care Surgery, Lunda/Aprahamian Chair Medical College of Wisconsin/Froedtert Trauma Center, Milwaukee, WI USA; 39 General Surgery, Letterkenny Hospital, Donegal, Ireland; 40 General Surgery, Centre Hospitalier Universitaire de Grenoble, Grenoble, France; 41 Unit of Digestive and HPB Surgery, CARE Department Henri Mondor University Hospital (AP-HP), Faculty of Medicine, University of Paris Est, Créteil, France; 42 The Medical Center of Aurora and Spalding Rehabilitation Hospital, Aurora, CO USA; 43 Chief, Trauma, Critical Care, Emergency Surgery, VCU Health System, Richmond, VA USA; 44 Surgery, Radbouc UMC, Nijmegen, The Netherlands; 45 Director of General Surgery Department, ASUR Marche, AV5, Hospital of San Benedetto del Tronto (AP) - Sapienza University, Rome, Italy; 46 Consultant Emergency Surgery, Professional Specialist at Ethiopian Air Force Hospital, President of Ethiopian Emergency Surgery Association, Bishoftu, Oromia Ethiopia; 47 Department of Surgery, Tianjin Nankai Hospital, Nankai Clinical School of Medicine, Tianjin Medical University, Tianjin, China; 48 Department of Traumatology, John Hunter Hospital and University of Newcastle, Newcastle, NSW Australia; 49 MedAlliance, Albury, NSW Australia; 50 Professor and Vice Chair of Surgery, LAC+USC Medical Center, University of Southern California, Los Angeles, CA USA

## Correction to: World Journal of Emergency Surgery (2022) 17:18 10.1186/s13017-022-00419-x

Following the publication of the original article [1], in html tagging the author name “Dimitris Damaskos” under WSES MIS working group was incorrectly written as “Dimitris Damascos”.

The original article has been corrected.
