# Supplementary material for: Minimally invasive surgery in emergency surgery: a WSES survey
Source: World J Emerg Surg. 2022 Mar 18;17:18. doi: 10.1186/s13017-022-00419-x (PMC8932166; doi:10.1186/s13017-022-00419-x)
Supplement: Supplementary file 1 — Additional file 1: Survey's questionnaire. [file 13017_2022_419_MOESM1_ESM.pdf]

Minimally Invasive Surgery in Emergency (MISE)

Dear Colleague,
the aim of the current online survey by the World Society of Emergency Surgery (www.wses.org.uk) is to explore the use of Minimally Invasive Surgery in Primary Surgical Emergencies and in Secondary Surgical Emergencies, which are emergency surgical conditions after elective Minimally Invasive Surgery.
Please take a few minutes to fill the questionnaire.

The results will be available on the World Journal of Emergency Surgery website (wjes.biomedcentral.com); moreover, a review focused on this topic will be published. If you wish to collaborate for further activities, please leave your e-mail in the dedicated box.
Please, also feel free share this questionnaire with as many colleagues as possible!

Thank you very much for your time!

*Required

1. Email *

To share the Questionnaire with your colleagues via social apps, please scan the QR code and forward it to your contacts

2. Please indicate your affiliation *

PERSONAL CHARACTERISTICS:

The aim of this section is to characterize the profile of the responder surgeon in order to better investigate the results of the present survey and describe the use of MIS in Emergency among surgeons with different subspecializations.

3. Please state your age? (NUMBER) *

____

4. Gender (M/F) *

Mark only one oval.

- ( ) Male
- ( ) Female
- ( ) Prefer not to say

5. How long is your professional experience as a surgeon? *

Mark only one oval.

- ( ) 0-5 years
- ( ) 6-15 years
- ( ) >15 years
- ( ) Resident / Registrar

6. Please rate the area of expertise for each subspecialty. *

Mark only one oval per row.

	0 - none	1	2	3	4	5 - high
General surgery	⬭	⬭	⬭	⬭	⬭	⬭
Emergency surgery and Trauma surgery	⬭	⬭	⬭	⬭	⬭	⬭
Colorectal surgery	⬭	⬭	⬭	⬭	⬭	⬭
Upper GI surgery	⬭	⬭	⬭	⬭	⬭	⬭
HPB surgery	⬭	⬭	⬭	⬭	⬭	⬭
Endocrine surgery	⬭	⬭	⬭	⬭	⬭	⬭
Bariatric surgery	⬭	⬭	⬭	⬭	⬭	⬭

7. Do you usually perform MIS (laparoscopy or robotic) for major elective abdominal surgery ? *

Mark only one oval per row.

	never	less than 25% of cases	25-50%of cases	more than 50% of cases
Laparoscopy	⬭	⬭	⬭	⬭
Robotic	⬭	⬭	⬭	⬭

8. Do you usually perform MIS (laparoscopy or robotic) for primary surgical emergencies? *

Mark only one oval per row.

	Never	Less than 25% of cases	25-50%of cases	More than 50% of cases
Laparoscopy				
Robotic				

9. Do you usually perform MIS (laparoscopy or robotic) for secondary surgical emergencies (complications of elective surgery)? *

Mark only one oval per row.

	Never	Less than 25% of cases	25-50%of cases	More than 50% of cases
Laparoscopy				
Robotic				

HOSPITAL CHARACTERISTICS:

The aim of this section is to provide information regarding the characteristics of the different institutions in which the responder of the survey practise.

10. In which country do you work? *

11. Is your hospital a public or a private institution? *

Mark only one oval.

- Public Hospital
- Private Hospital

12. Is your hospital a first-, second- or third-level institution? *

Mark only one oval.

- First-level (rural/community hospital)
- Second-level (regional/general hospital)
- Third-level (academic/referral hospital)

13. Is there an Emergency Department at your hospital? *

Mark only one oval.

- Yes
- No

14. Is there a dedicated Emergency Surgery / Acute Care Surgery Unit at your hospital? *

Mark only one oval.

- Yes
- No

15. Which proportion of your current practice is dedicated to emergency surgery? *

Tick all that apply.

- 0%
- 1-25%
- 26-50%
- >50%

INDICATION FOR THE USE OF MISE

The aim of this section is to explore your confidence in managing primary and secondary surgical emergencies

16. Please rate the level of your personal confidence with MISE for the following primary emergencies *

Mark only one oval per row.

	I think it is not indicated	0 - none	1	2	3	4	5 - high
Acute Appendicitis							
Acute Calculous Cholecystitis							
Perforation of Gastric and Duodenal Ulcer							
Acute Diverticulitis							
Bowel Obstruction due to peritoneal adhesions							
Oesophageal perforation							
Iatrogenic Upper or Lower GI perforation							
Abdominal exploration							
Strangulated Ventral Hernia							
Necrosectomy for Infected Acute Pancreatic Necrosis							

17. If you believe an emergency surgical procedure is not described above, please specify and rate (0 to 5) your confidence with MISE with this supplementary primary emergency procedure

____

18. Please rate the level of your personal confidence with MISE for the following secondary emergencies *

Mark only one oval per row.

	I think it is not indicated	0 - none	1	2	3	4	5 - high
Dehiscence of intestinal anastomosis (included colorectal anastomosis)	◯	◯	◯	◯	◯	◯	◯
Dehiscence of gastro intestinal anastomosis after partial gastric resection	◯	◯	◯	◯	◯	◯	◯
Bile leak after cholecystectomy	◯	◯	◯	◯	◯	◯	◯
Bile leak after liver resection	◯	◯	◯	◯	◯	◯	◯
Postoperative haemoperitoneum	◯	◯	◯	◯	◯	◯	◯
Postoperative bowel obstruction	◯	◯	◯	◯	◯	◯	◯

19. If you believe an emergency surgical procedure is not described above, please specify and rate (0 to 5) your confidence with MISE with this supplementary secondary emergency procedure

____

LIMITATIONS IN THE USE OF MISE

The aim of this section is to explore the limits in approaching primary and secondary emergencies with MIS.

LIMITS in WORKING CONDITIONS

Please rate the relevance of the following items in limiting a MIS approach to primary and secondary emergencies. The emergercy surgical procedures are divided into four groups.

20. LIMITS in GROUP 1: Appendicitis, Cholecystitis *

Mark only one oval per row.

	0 - none	1	2	3	4	5 - high
Your own surgical skills	◯	◯	◯	◯	◯	◯
Nursing skills	◯	◯	◯	◯	◯	◯
Night-time operation	◯	◯	◯	◯	◯	◯
Technology availability	◯	◯	◯	◯	◯	◯
Extimated prolonged surgical duration	◯	◯	◯	◯	◯	◯

21. LIMITS in GROUP 2: Perforation of Gastric and Duodenal Ulcer, Bowel Occlusion due to peritoneal adhesions *

Mark only one oval per row.

	0 - none	1	2	3	4	5 - high
Your own surgical skills						
Nursing skill						
Night-time operation						
Technology availability						
Extimated surgical duration						

22. LIMITS in GROUP 3: Colon resection for Hinchey 3 and 4 Acute Diverticulitis *

Mark only one oval per row.

	0 - none	1	2	3	4	5 - high
Your own surgical skills	⬭	⬭	⬭	⬭	⬭	⬭
Nursing skill	⬭	⬭	⬭	⬭	⬭	⬭
Night-time operation	⬭	⬭	⬭	⬭	⬭	⬭
Technology availability	⬭	⬭	⬭	⬭	⬭	⬭
Extimated prolonged surgical duration	⬭	⬭	⬭	⬭	⬭	⬭

23. LIMITS in GROUP 4: Dehiscence of intestinal anastomosis (included colorectal anastomosis), dehiscence of gastrointestinal anastomosis after partial gastectomy, bile leak after cholecystectomy, bile leak after liver resection, postoperative haemoperitoneum, postoperative intestinal obstruction *

Mark only one oval per row.

	0 - none	1	2	3	4	5 - high
Your own surgical skills	◯	◯	◯	◯	◯	◯
Nursing skill	◯	◯	◯	◯	◯	◯
Night-time operation	◯	◯	◯	◯	◯	◯
Technology availability	◯	◯	◯	◯	◯	◯
Extimated prolonged surgical duration	◯	◯	◯	◯	◯	◯

LIMITS in CLINICAL CONDITIONS

Please rate the relevance of the following items in limiting a MIS approach to primary and secondary emergencies. The emergercy surgical procedures are divided into four groups.

24. LIMITS in GROUP 1: Appendicitis, Cholecystitis *

Mark only one oval per row.

	0 - none	1	2	3	4	5 - high
Shock condition	◯	◯	◯	◯	◯	◯
Age	◯	◯	◯	◯	◯	◯
ASA score	◯	◯	◯	◯	◯	◯
APACHE score	◯	◯	◯	◯	◯	◯
PPossum	◯	◯	◯	◯	◯	◯
ACS NSQIP Surgical Risk	◯	◯	◯	◯	◯	◯
Previous abdominal surgery	◯	◯	◯	◯	◯	◯

25. LIMITS in GROUP 2: Perforation of Gastric and Duodenal Ulcer, Bowel Occlusion due to peritoneal adhesions *

Mark only one oval per row.

	0 - none	1	2	3	4	5 - high
Shock condition	◯	◯	◯	◯	◯	◯
Age	◯	◯	◯	◯	◯	◯
ASA score	◯	◯	◯	◯	◯	◯
APACHE score	◯	◯	◯	◯	◯	◯
PPossum	◯	◯	◯	◯	◯	◯
ACS NSQIP Surgical Risk	◯	◯	◯	◯	◯	◯
Previous abdominal surgery	◯	◯	◯	◯	◯	◯

26. LIMITS in GROUP 3: Colon resection for Hinchey 3 and 4 Acute Diverticulitis *

Mark only one oval per row.

	0 - none	1	2	3	4	5 - high
Shock condition	⬭	⬭	⬭	⬭	⬭	⬭
Age	⬭	⬭	⬭	⬭	⬭	⬭
ASA score	⬭	⬭	⬭	⬭	⬭	⬭
APACHE score	⬭	⬭	⬭	⬭	⬭	⬭
PPossum	⬭	⬭	⬭	⬭	⬭	⬭
ACS NSQIP Surgical Risk	⬭	⬭	⬭	⬭	⬭	⬭
Previous abdominal surgery	⬭	⬭	⬭	⬭	⬭	⬭

27. LIMITS in GROUP 4: Dehiscence of intestinal anastomosis (included colorectal anastomosis), Dehiscence of gastro intestinal anastomosis after partial gastric resection, Bile leak after cholecystectomy, Bile leak after liver resection, post operative Hemoperitoneum, Post operative occlusion *

Mark only one oval per row.

	0 - none	1	2	3	4	5 - high
Shock condition	⬭	⬭	⬭	⬭	⬭	⬭
Age	⬭	⬭	⬭	⬭	⬭	⬭
ASA score	⬭	⬭	⬭	⬭	⬭	⬭
APACHE score	⬭	⬭	⬭	⬭	⬭	⬭
PPossum	⬭	⬭	⬭	⬭	⬭	⬭
ACS NSQIP Surgical Risk	⬭	⬭	⬭	⬭	⬭	⬭
Previous abdominal surgery	⬭	⬭	⬭	⬭	⬭	⬭

LIMITATION IN THE PROSECUTION OF MISE

The aim of this section is to explore the reasons of conversions from MIS to a more invasive approach in primary and secondary surgical emergencies.

LIMITS to PROSECTUION

Please rate the relevance of the following items as reasons for conversion to laparotomy. The emergercy surgical procedures are divided into four groups.

28. GROUP 1: Appendicitis, Cholecystitis *

Mark only one oval per row.

	0 - none	1	2	3	4	5 - high
Duration of the surgical procedure	◯	◯	◯	◯	◯	◯
Bleeding	◯	◯	◯	◯	◯	◯
Unclear/suboptimal visualization of anatomical structures	◯	◯	◯	◯	◯	◯
Bowel perforation	◯	◯	◯	◯	◯	◯
Intraoperative clinical deterioration	◯	◯	◯	◯	◯	◯

29. GROUP 2: Perforation of Gastric and Duodenal Ulcer, Bowel Occlusion due to peritoneal adhesions *

Mark only one oval per row.

	0 - none	1	2	3	4	5 - high
Duration of the surgical procedure	◯	◯	◯	◯	◯	◯
Bleeding	◯	◯	◯	◯	◯	◯
Unclear/suboptimal visualization of anatomical structures	◯	◯	◯	◯	◯	◯
Perforation	◯	◯	◯	◯	◯	◯
Intraoperative clinical deterioration	◯	◯	◯	◯	◯	◯

30. GROUP 3: Colon resection for Hinchey 3 and 4 Acute Diverticulitis *

Mark only one oval per row.

	0 - none	1	2	3	4	5 - high
Duration of the surgical procedure	⬭	⬭	⬭	⬭	⬭	⬭
Bleeding	⬭	⬭	⬭	⬭	⬭	⬭
Unclear/suboptimal visualization of anatomical structures	⬭	⬭	⬭	⬭	⬭	⬭
Perforation	⬭	⬭	⬭	⬭	⬭	⬭
Intraoperative clinical deterioration	⬭	⬭	⬭	⬭	⬭	⬭

31. GROUP 4: Dehiscence of intestinal anastomosis (included colorectal anastomosis), Dehiscence of gastro intestinal anastomosis after partial gastric resection, Bile leak after cholecystectomy, Bile leak after liver resection, post operative Hemoperitoneum, Post operative occlusion *

Mark only one oval per row.

	0 - none	1	2	3	4	5 - high
Duration of the surgical procedure	◯	◯	◯	◯	◯	◯
Bleeding	◯	◯	◯	◯	◯	◯
Unclear/suboptimal visualization of anatomical structures	◯	◯	◯	◯	◯	◯
Perforation	◯	◯	◯	◯	◯	◯
Intraoperative clinical deterioration	◯	◯	◯	◯	◯	◯

Perspective in MISE (optional)

Would do you like to share your opinion and/or suggestions about the application of MIS in emergency surgery?
Please write your suggestions regarding research perspectives and/or your personal opinion.
Thank you!

32. Primary Emergency (PE) - excluding trauma

33. Intraoperative Surgical Emergency Maneuvers (ISEM)

34. Secondary Emergency (SE): reintervention after elective minimally invasive surgery (EMIS)

To share the Questionnaire with your colleagues via social apps, please scan the QR code and forward it to your contacts

This content is neither created nor endorsed by Google.

Google Forms
