# Supplementary material for: Minimally invasive surgery in emergency surgery: a WSES survey
Source: World J Emerg Surg. 2022 Mar 18;17:18. doi: 10.1186/s13017-022-00419-x (PMC8932166; doi:10.1186/s13017-022-00419-x)
Supplement: Supplementary file 2 — Additional file 2: Members of the WSES MIS Consortia. [file 13017_2022_419_MOESM2_ESM.docx]

| Family Name | Name | Affiliation |
| --- | --- | --- |
| Adamovich | Dmitry M. | Gomel State Medical University; Location: Gomel, Belarus; Department of Surgical Diseases II, (general surgery) |
| Agastra | Ervis | General Surgery Clinic, Regional Hospital of Korce, Albania |
| Agresta | Ferdinando | Department of General Surgery, AULSS 2 Trevigiana, Vittorio Veneto (TV), Italy |
| Akın | Emrah | Sakarya University Educational and Research Hospital |
| Akkapulu | Nezih | Hacettepe University, ANkara;Turkey |
| Ali | Syed MUHAMMAD | Hamad Medical Corporation, Doha, Qatar |
| Amaya Luna Victoria | Renzo | Medico |
| Ammendola | Michele | Health of Science Department, "Magna Graecia" University Medical School, "Mater Domini" Hospital, Digestive Surgery Unit, Catanzaro, Italy |
| Ammerata | Giorgio | Università degli Studi Magna Graecia di Catanzaro |
| Angel Diaz | Angel Diaz | Advance laparoscopic surgeon |
| Angelico | Roberta | HPB and Transplant Unit, Department of Surgical Sciences, University of Rome Tor Vergata |
| Antonella | Delvecchio | UOC CHIRURGIA GENERALE "A. PERRINO" BRINDISI |
| Aprea | Giovanni | Università degli studi Federico II di Napoli |
| Armellino | Mariano Fortunato | AOU San Giovanni di Dio e Ruggi d'Aragona - Salerno |
| Arroyo Murillo | Gabriela Aracelly | General Surgery and Minimally Invasive Unit of Dolo Hospital, Ulss Serenissima |
| Askevold | Ingolf | University of Giessen, Department for General Surgery |
| Atanasov | Boyko | UMHAT EUROHOSPITAL, Medical university of Plovdiv Bulgaria |
| Augustin | Goran | University Hospital Centre Zagreb |
| Awad | Selmy | Mansoura university hospital , Mansoura university |
| Baili | Efstratia | NKUA (National and Kapodistrian University of Athens) |
| Bains | Lovenish | Maulana Azad Medical College, New Delhi, India |
| Bairwa | Banwari Lal | Chief surgeon, MP Birla hospital and research centre, chittorgarh, INDIA |
| Bala | Miklosh | Faculty of Medicine, Hadassah Hebrew University, General Surgery and Trauma Department, Jerusalem, Israel |
| Baldini | Edoardo | ASST Melegnano |
| Balestra | Francesco | Policlinico Sassarese |
| Barberis | Andrea | E.O. Ospedali Galliera, Genova |
| Barone | Mirko | Department of Surgery, SS. Annunziata University Hospital of Chieti (Italy) |
| Baronio | Gianluca | Ospedale di Esine |
| Bekele | Kebebe | Surgery Department, Maddawalabu University Goba Referral Hospital |
| Bellanova | Giovanni | D. Camberlingo Hospital Francavilla Fontana (BR) |
| BELTRAN | MARCELO | HOSPITAL DE LA SERENA |
| Beltran Miranda | Pablo | University Hospital Juan Ramon Jimenez |
| Benedetti | Antonio | Università degli Studi di Milano Statale |
| Ben-Ishay | Offir | Surgical Oncology Service, Department of General Surgery, Rambam Health Care Campus |
| Bernardi | Daniele | Università degli studi di milano |
| Bidarahalli Krishna | Prasanna | Sapthagiri Institute of Medical Sciences and Research Center |
| Bondurri | Andrea | Luigi Sacco Univerity Hospital |
| Borasi | Andrea | Humanitas Gradenigo, Torino |
| Brillantino | Antonio | Department of Emergency Surgery, “A. Cardarelli” hospital, Naples, Italy |
| Brisinda | Giuseppe | Fondazione Policlinico Universitario A Gemelli IRCCS |
| Buonomo | Luis | Director of Emergency, Urgency and Trauma; School of Medicine-University of Buenos Aires; ARGENTINA |
| Cahill | Ronan | UCD Centre for Precision Surgery, University College Dublin, Dublin, Ireland |
| Campanelli | Michela | Emergency Surgery Unit, University Hospital of Tor Vergata, viale oxford 81, 00133 Rome |
| Carlos | Fretel | Si |
| Carrano | Francesco Maria | University of Rome Tor Vergata |
| Casoni Pattacini | Gianmaria | Chirurgia generale,d’urgenza e nuove tecnologie Ospedale Civile di Baggiovara Modena |
| Castaldi | Antonio | University of Naples Federico II |
| Castaldo | Pasquale | AOPC Catanzaro |
| Chouliaras | Christos | MD |
| Chowdhury | Sharfuddin | King Saud Medical City, Riyadh, Saudi Arabia |
| Cianci | Pasquale | Lorenzo Bonomo Hospital-ASL BAT |
| Cioffi | Stefano Piero Bernardo | General Surgery - Trauma Team, ASST GOM Niguarda, Milano |
| Clarizia | Guglielmo | General Surgery unit, asst valtellina alto lario, hospital of sondrio |
| Lopes Moreira | Claudia Cristina | General surgeon |
| Cobianchi | Lorenzo | University of Pavia. IRCCS Policlinico San Matteo. Pavia.Italy. |
| Cocuz | Iuliu Gabriel | Pathophysiology Department, George Emil Palade University of Medicine, Pharmacy, Sciences and Technology of Targu Mures, Romania |
| Coimbra | Raul | Riverside University Health System / Loma Linda University |
| Colak | Elif | University of Samsun |
| Conti | Luigi | Acute care Surgery Unit , Department of Surgery, AUSL Piacenza |
| Cordeiro Fonseca | Vinicius | Hospital ViValle |
| Correia de Sá | Tiago | Centro Hospitalar do Tâmega e Sousa |
| Cosci | Marco | AULSS 8 BERICA Vicenza Chirurgia Generale |
| Costa | Sílvia | CHVNG/E, EPE |
| Currò | Giuseppe | Surgical Unit, Health Science Department, University Magna Graecia of Catanzaro, Catanzaro, Italy |
| D'Acapito | Fabrizio | Ospedale Morgagni-Pierantoni, Forlì AUSL Romagna |
| Davies | Justin | Addenbrooke’s Hospital, Cambridge |
| Dawe | Jean Philip | Trauma surgeon Vancouver General Hospital |
| De Andrés-Asenjo | Beatriz | Hospital Clínico Universitario de Valladolid (Spain) |
| De Simoni | Ottavia | IOV, Chirurgia oncologica delle vie digestive |
| Deida | Simona | Department of surgical science, university of cagliari |
| Demetrashvili | Zaza | Associate Professor, Surgery Department, Tbilisi State Medical University. |
| Demirli Atici | Semra | University of Health Sciences Tepecik Training and Research Hospital, Department of General Surgery |
| Demma | Jonathan Abraham | Hadassah medical center Hebrew university of Jerusalem |
| Detanac | Dzemail | General hospital Novi Pazar, Novi Pazar, Serbia |
| Di Maggio | Francesco | Barking Havering and Redbridge University Hospital NHS Trust |
| Di Martino | Marcello | A.O.R.N. Cardarelli |
| Dimitrov | Evgeni | Department of Surgical Diseases, University Hospital "Prof. Dr. Stoyan Kirkovich" Stara Zagora, Bulgaria |
| Dinuzzi | Vincenza Paola | Department of Clinical Medicine and Surgery, Federico II University of Naples, Sergio Pansini 5, 80131, Naples, Italy. |
| Dubuisson | Vincent | Bordeaux University Hospital |
| Dziakova | Jana | Hospital Clínico San Carlos |
| Ebrahim | Mohamed | Gastrounit, Surgical Division, Hvidovre, Copenhagen University Hospital |
| Elberm | Hassan | University Hospital Southampton |
| Ernisova | Mairam | Kyrgyz State Medical academy after I.K. Achunbaev |
| Estaire-Gomez | Mercedes | Dra. |
| Favi | Francesco | Chirurgia Generale e d'Urgenza - Ospedale M. Bufalini CESENA |
| Ferrara | Francesco | Department of Surgery, San Carlo Borromeo Hospital, ASST Santi Paolo e Carlo, Milan, Italy |
| Ferrario | Luca | University of Milan |
| Ferreres | Alberto | University of Buenos Airrs |
| Fleres | Francesco | General Surgery Unit - ASST Valtellina e Alto Lario, Sondrio Hospital - Sondrio - Italy |
| Francesk | Mulita | Department of Surgery, General University Hospital of Patras |
| Frazzetta | Giuseppe | ARNAS Civico Palermo chirurgia oncologica |
| Frigerio | Isabella | PEDERZOLI HOSPITAL |
| Frontali | Alice | General Surgery Unit, ASST Brianza, Vimercate Hospital, Via Santi Cosma e Damiano, 10, 20871, Vimercate, Italy |
| Gachabayov | Mahir | Vladimir City Emergency Hospital |
| Galatioto | Christian | Emergency surgery unit, azienda ospedaliero universitaria pisana |
| Gandhi | Chinmay | Professor |
| Gargarella | Simone | University Department of Medical, Oral and Biotechnological Sciences, Gabriele d'Annunzio University of Chieti-Pescara, Chieti, Italy |
| Garulli | Gianluca | Azienda Romagna |
| Garzali | Ibrahim umar | Aminu Kano Teaching Hospital kano Nigeria |
| Gatti | Matteo | Chirurgia Generale , ASL NO |
| Ghannam | Wagih | Mansoura faculty of medicine |
| Ghazi | Faiz Najmuddin | Hospital Universiti Sains Malaysia |
| Ghignone | Federico | General and colorectal surgery unit, Ospedale Santa Maria delle Croci (Ravenna, Italy) |
| Giordano | Marco | U.O. Chirurgia Generale- Ospedale Infermi- Rimini |
| Giraudo | Giorgio | Department of Surgery ASO Santa Croce e Carle CUNEO |
| Giuffrida | Mario | Parma University Hospital General Surgery Unit |
| Gomes | Carlos Augusto Gomes | NIPU (NUCLEO INTERDISCIPLINAR DE PESQUISA EM UROLOGIA) UNIVESIDADE FEDERAL DE JUIZ DE FORA - UFJF - BRASIL |
| Gomez-Sandoval | Moises | Surgical Oncology |
| Guariniello | Anna | Ospedale Santa Maria delle Croci, Ravenna |
| Gudisa | Zewdie | Addis Ababa university |
| Gunadi | . | Faculty of Medicine, Public Health and Nursing, Universitas Gadjah Mada |
| Guner | Ali | Karadeniz Technical University, Faculty of Medicine |
| Gupta | Sanjay | Government Medical College and Hospital Chandigarh India |
| Hamid | Hytham K. S | Kuwaiti Specialized Hospital |
| Hardcastle | Timothy Craig | University of KwaZulu-Natal and Dept of Health KZN |
| Hecker | Andreas | Dept. of General & Thoracic Surgery, University Hospital of Giessen, Germany |
| Inama | Marco | General Surgery Department - Pederzoli Hospital |
| Ioannidis | Orestis | 4th Department of Surgery, Medical School, Aristotle University of Thessaloniki, General Hospital “George Papanikolaou”, Thessaloniki, Greece |
| Kafka | Reinhold | Medical University Innsbruck |
| Kao | Lillian | McGovern Medical School at UTHealth |
| Kara | Yasin | Health Sciences University Kanuni Sultan Süleyman Training and Research Hospital General Surgery Clinic |
| kavalakat | Alfie J. | Jubilee Mission Medical College & RI, Thrissur |
| Kechagias | Aristotelis | Department of Digestive Surgery, Kanta-Häme Central Hospital, Finland |
| Khokha | Vladimir | General surgery Dept., Mozyr City hospital, Belarus |
| Kim | Jae Il | Department of Surgery, Ilsan Paik Hospital, Inje University College of Medicine, Goyang, Republic of Korea |
| Kirkby-Bott | James | University Hospital Southampton, UK |
| Kirkpatrick | Andrew Wallace Kirkpatrick | University of Calgary, Calgary, Alberta |
| Kobe | Yoshiro | Chiba Emergency Medical Center |
| Kok | Kenneth Y. Y. | Pengiran Anak Puteri Rashidah Sa'adatul Bolkiah Institute of Health Sciences, Universiti Brunei Darussalam |
| Komaei | Iman | General Surgery Unit, University Teaching Hospital "G. Martino" of Mesina |
| KONG | VICTOR | Department of Surgery, University of KwaZulu Natal, Durban, South Africa |
| Korkolis | Dimitris | Hellenic Anticancer Hospital “Saint Savvas” |
| Kössi | Jyrki | Päijät-Häme Central Hospital |
| Koukoulis | Georgios | General Hospital of Larisa |
| Kryvoruchko | Igor A. | Head of Department of Surgery No 2. Kharkiv National Medical University, Ukraine. |
| Kuriyama | Akira | Emergency and Critical Care Center, Kurashiki Central Hospital |
| Lali | Akhmeteli | Tbilisi State Medical University, Department of Surgery |
| Landaluce-Olavarria | Aitor | Urduliz hospital(Osakidetza- Vizcaya) |
| Leão | Pedro | General Surgery, Colorectal Unit, Grupo Trofa Saude, Braga, Portugal. |
| Lee | Jae Gil | Department of Surgery, Yonsei University College of Medicine |
| Licari | Leo | Department of Surgical, Oncological and Oral Sciences - University of Palermo |
| Lisi | Giorgio | Sant’Eugenio Hospital, Rome, Italy |
| Lizarazu | Aintzane | General surgeon |
| Lohsiriwat | Varut | Department of Surgery, Faculty of Medicine Siriraj Hospital, Mahidol University, Bangkok, Thailand |
| Lopez-Lopez | Victor | Clinic and University Virgen de la Arrixaca Hospital. IMIB-ARRIXACA |
| Lopez-Ruiz | Jose Antonio | Hospital Universitario Virgen Macarena |
| Luppi | Davide | General And Emergency Surgery ASMN IRCCS Reggio Emilia |
| Machain Vega | Gustavo Miguel | Hospital de Clinicas. Facultad de Ciencias Medicas Universidad Nacional de Asunción. Paraguay |
| Machairas | Nikolaos | 2nd Department of Propaedeutic Surgery, General Hospital of Athens “Laiko”, Athens, Greece |
| Maegele | Marc | Cologne-Merheim Medical Center (CMMC), Department of Trauma and Orthopedic Surgery, Institute for Research in Operative Medicine (IFOM), University Witten/Herdecke, Campus Cologne-Merheim, Cologne (Germany) |
| Major | Piotr | Jagiellonian University Medical College |
| Malhotra | Ravinder singh | SARVHIT GASTROCITY |
| Manatakis | Dimitrios | Department of Surgery, Athens Naval and Veterans Hospital, Athens, Greece |
| Mantoglu | Baris | Sakarya University Training and Research Hospital |
| Mariani | Nicoló Maria | ASST Santi Paolo e Carlo, Milano (Italy) |
| Martines | Gennaro | Azienda Ospedaliero Universitaria Policlinico Bari - Italy |
| Martínez-Pérez | Aleix | Department of General and Digestive Surgery. Hospital Universitario Doctor Peset. Valencia (SPAIN). |
| Massalou | Damien | Centre Hospitalier Universitaire de Nice, France |
| Medappil | Noushif | Consultant - GI, HPB and Liver transplant surgery, Aster MIMS, Calicut, India |
| MEDJMADJ | Nacer | c |
| Menegozzo | Carlos Augusto Metidieri | Division of General Surgery and Trauma, University of Sao Paulo |
| Meriç | Serhat | wses |
| Mesina | Cristian | Emergency County Hospital of Craiova, University of Medicine and Pharmacy of Craiova |
| Mihailescu | Andrei | Tameside and Glossop Integrated Care NHSFT |
| Milone | Marco | University of Naples Federico II |
| Mishra | Tushar S | All India Institute of Medical Sciences Bhubaneswar |
| Mohamedahmed | Ali Yasen Y. | SWBH NHS trust - UK |
| Mohan | Rajashekar | All India Institute of Medical Sciences, Mangalagiri, Andhra Pradesh, India |
| Monika Gureh | Dr Monika Gureh | Senior resident |
| Montuori | Mauro | Department of Surgery, Policlinico San Pietro, Ponte San Pietro, Italy |
| Morales Morales | Diana | IMSS |
| Mukherjee | Indraneil | Staten Island University Hospital |
| Munyika | Akutu | Onandjokwe Hospital Namibia |
| Musina | Ana-Maria | University of Medicine and Pharmacy Grigore T. Popa, Surgery Department, Iasi, Romania |
| Naidu | Krishanth | Concord Repatriation General Hospital |
| Nashidengo | Pueya Abdulrashid | Windhoek Central Academic Hospital |
| Ndong | Abdourahmane | Gaston Berger University, Saint-Louis, Sénégal |
| Negoi | Ionut | Carol Davila University of Medicine and Pharmacy Bucharest, Emergency Hospital of Bucharest, Romania |
| Nikolopoulos | Ioannis | Lewisham & Greenwich NHS Trust |
| Nita | Gabriela | Sant’Anna Hospital, AUSL Reggio Emilia |
| Olmi | Stefano | Policlinico San Marco, Zingonia (Bg) |
| Omoshoro-Jones | Jones | CHBAH/Wits |
| Oshevire-Bini | Charles Aghogho | King Khalid General Hospital |
| Pal | Ajay Kumar | King George’s Medical University |
| Palini | Gian Marco | Ospedale infermi rimini |
| Palomba | Giuseppe | Università degli studi di Napoli Federico II |
| Pantalone | Desirè | UNIVERSITY OF FLORENCE DEPT OF EXPERIMENTAL AND CLINICAL MEDICINE |
| Pantoja Pachajoa | Diana Alejandra | Clinica Universitaria Reina Fabiola |
| Panyko | Arpád | University Hospital Bratislava and Faculty of Medicine, Comenius University Bratislava |
| Pararas | Nikolaos | Dr Sulaiman Al Habib Hospital |
| Parreira | Jose Gustavo | Faculdade de Ciências Médicas da Santa Casa de São Paulo |
| Pata | Francesco | Nicola Giannettasio Hospital, Corigliano-Rossano, Italy |
| Patiti | Mauro | Acoi |
| Pellino | Gianluca | Università degli Studi della Campania "Luigi Vanvitelli" |
| Perivoliotis | Konstantinos | General Hospital of Volos |
| Perra | Teresa | Università degli Studi di Sassari, Italia |
| Perrone | Gennaro | Department of emergency Surgery, Parma Maggiore Hospital, Parma, Italy |
| Pesce | Antonio | Azienda USL of Ferrara-University of Ferrara |
| Pilavas | Andreas | n/a |
| Pinotti | Enrico | Policlinico San Pietro |
| Pintar | Tadeja | UMC Ljubljana |
| Podda | Mauro | Department of Surgical Science, University of Cagliari, Italy |
| Porcu | Alberto | Università degli Studi di Sassari, Italia |
| Rahim | Razrim | Universiti Sains Islam Malaysia, Malaysia. |
| Rahman Mitul | Ashrarur | Bangladesh Shishu Hospital & Institute |
| Reichert | Martin | Department of General, Visceral, Thoracic, Transplant and Pediatric Surgery, University Hospital of Giessen, Germany |
| Rezende-Neto | Joao | St. Michael's Hospital University of Toronto |
| Ribeiro Jr | Marcelo A. F. | Department of Surgery Catholic university of São Paulo - Sorocaba |
| Robustelli | Umberto | A.O.R.N A Cardarelli - Napoli |
| Rodriguez | Agustin | Professor |
| Romeo | Luigi | Chirurgia d'urgenza, Arcispedale Sant'Anna di Ferrara |
| Rosa | Fausto | Fondazione Policlinico Universitario A. Gemelli IRCCS |
| Roscio | Francesco Pietro Maria | ASST Valle Olona, Busto Arsizio |
| Rossi | Stefano | Emergency surgery San Filippo Neri hospital ASL Roma1 |
| Runfola | Matteo | ARNAS "G Brotzu" |
| Rutegård | Martin | Surgical and Perioperative Sciences, Surgery, Umeå University, Umeå, Sweden |
| Sall | Ibrahima | Department of general surgery, military teaching hospital, Hôpital Principal de Dakar. |
| Sammartano | Fabrizio | Trauma Unit, General Surgery, San Carlo Borromeo Trauma Center, Milan |
| Santarelli | Mauro | Città della Salute e della Scienza |
| Sasia | Diego | Santa Croce and Carle Hospital, Cuneo |
| Sawyer | Robert | Western Michigan University Homer Stryker MD School of Medicine |
| Schizas | Dimitros | FIRST DEPARTMENT OF SURGERY, NATIONAL AND KAPODISTRIAN UNIVERSITY OF ATHENS, LAIKON GENERAL HOSPITAL, ATHENS, GREECE |
| Schneck | Anne-Sophie | CHU Guadeloupe |
| Seretis | Charalampos | General University Hospital of Patras, Greece |
| Serrablo | Alejandro | Chairman of HPB Surgery |
| Serradilla-Martín | Mario | Instituto de Investigación Sanitaria Aragón. Department of Surgery. Miguel Servet University Hospital, Zaragoza, Spain |
| Shelat | Vishal | Tan Tock Seng Hospital, Singapore |
| Sibilla | Maria Grazia | Università degli studi di Ferrara |
| Siragusa | Leandro | Department of surgery, Università degli studi di Roma Tor Vergata |
| Slavchev | Mihail | University Hospital Eurohospital - Plovdiv |
| Soggiu | Fiammetta | London North West University Healthcare NHS Trust |
| Solaini | Leonardo | Department of Medical and Surgical Sciences, University of Bologna, Morgagni-Pierantoni Hospital, Forlì |
| Soldini | Gabriele | Ospedale Fatebenefratelli sacra famiglia Erba |
| Somigli | Riccardo | usl toscana centro- santa maria nuova |
| Sopuev | Andrey | Kyrgyz State Medical Academy named after I.K. Akhunbaeva. Head of the Department of Hospital Surgery with the Course of Operative Surgery |
| Sotiropoulou | Maria | Evaggelismos Hospital |
| Spolini | Alessandro | ASST VALTELLINA ALTO LARIO |
| Sydorchuk | Ruslan | Regional A & E Hospital Chernivtsi, Ukraine |
| Sydorchuk | Larysa | Bukovinian State Medical University, Ukraine |
| Tarasconi | Antonio | Emergency Surgery Department, Maggiore Hospital of Parma, University of Parma, Italy |
| Tenreiro | Nádia | CHTMAD |
| Tiberio | Guido A.M. | Clinica Chirurgica Università di Brescia |
| Tolonen | Matti | Abdominal Center, Helsinki University Hospital and University of Helsinki, Finland |
| Tomadze | Gia | Tbilisi State Medical University |
| Tomajer | Valentina | Ospedale San Raffaele Milan |
| Toro | Adriana | General Surgery, Augusta Hospital, (SR) Italy |
| Trostchansky | Julio | Hospital Maciel.Uruguay |
| Tsekouras | Konstantinos | Sismanogleio General Hospital, Athens, Greece |
| Turrado-Rodriguez | Victor | Gastrointestinal Surgery Department, Hospital Clinic de Barcelona (Spain) |
| Tutino | Roberta | Chirurgia 1 - Treviso regional Hospital- ULSS2 Marca Trevigiana |
| Uccelli | Matteo | San Marco Hospital GSD - Zingonia (BG) - Italy |
| Ukkonen | Mika | Tampere University Hospital |
| Vailas | Michail | Laiko General Hospital |
| Cozza | Valerio | Fondazione Policlinico Universitario A Gemelli IRCCS |
| van Ramshorst | Gabrielle H. | Ghent University Hospital |
| Vasilescu | Alin | Department of Surgery, St Spiridon University Hospital, Grigore T Popa University of Medicine Iasi, Romania |
| Vega-Rivera | Felipe | Hospital Angeles Lomas |
| Vereczkei | Andras | Department of Surgery Clinical Center Medical School University of Pécs |
| Veroux | Massimiliano | Department of Medical and Surgical Sciences, University of Catania |
| VILALLONGA | RAMON | Bariatric and robotic department. Universitary Hospital Vall Hebron. Universitat Autònoma de Barcelona. Barcelona. Spain |
| Visconti | Diego | AOU Città della Salute e della Scienza di Torino - Chirurgia Generale d'Urgenza e PS |
| Walędziak | Maciej | Department of General, Oncological, Metabolic and Thoracic Surgery, Military Institute of Medicine, Szaserów 128 St., Warsaw, Poland. |
| Wannatoop | Tongporn | Faculty of Medicine Siriraj hospital, Mahidol university, Thailand |
| Widmer | Lukas Werner | Department of Visceral Surgery and Medicine, University Hospital Bern, Switzerland |
| Zakaria | Andee Dzulkarnaen | School of Medical Sciences & Hospital USM, Universiti Sains Malaysia, Kubang Kerian, Kelantan, Malaysia |
| Zanini | Nicola | AUSL Romagna |
| Zarnescu | Narcis Octavian | University Emergency Hospital Bucharest Romania |
| Zese | Monica | ULSS5 Polesana |
| Zuluaga | Mauricio | General and mis SURGEON |
